# Primary Splenic Classic Hodgkin Lymphoma with Immune Thrombocytopenic Purpura and Extramedullary Hematopoiesis

**DOI:** 10.1177/10668969261422261

**Published:** 2026-03-31

**Authors:** Ibrahim Elsharawi, Luke Y.C. Chen, Shannon Murphy, Tish O’Reilly, Sorin Selegean, Allam Shawwa

**Affiliations:** 1 Department of Pathology and Laboratory Medicine, Division of Hematological Pathology, 3688 Dalhousie University, Halifax, NS, Canada; 2 Division of Hematology, 12361 Dalhousie University, Halifax, NS, Canada; 3 Coastal Rare Inflammatory Diseases Program, 12361 Dalhousie University, Halifax, NS, Canada; 4 Division of Hematology, University of British Columbia, Vancouver, BC, Canada

**Keywords:** splenic classic hodgkin lymphoma, splenic lymphoma, classic hodgkin lymphoma, CHL, hodgkin lymphoma, immune thrombocytopenic purpura, refractory immune thrombocytopenic purpura, ITP, extramedullary hematopoiesis

## Abstract

**Background:**

Primary lymphomas of the spleen are rare, with primary splenic classic Hodgkin lymphoma (CHL) accounting for a minuscule fraction of these tumors, rendering it an exceptionally uncommon entity. Immune thrombocytopenic purpura (ITP) has been rarely reported in association with nodal CHL, and to our knowledge, there are no prior reports linking it with splenic CHL. Presented is an example of primary splenic CHL associated with ITP and extramedullary hematopoiesis (EMH), highlighting this unusual presentation of CHL.

**Patient presentation:**

A 70-year-old man presented with severe epistaxis and wet purpura in the mouth and low platelets. He had noted night sweats and fever for three months prior to presentation and had chronic mild thrombocytopenia. Imaging studies showed splenomegaly with scattered hypodensities in the spleen but no lymphadenopathy. The initial clinical suspicion based on imaging results was an indolent lymphoma. He was treated with IV immunoglobulin, rituximab, steroids and eltrombopag with no response. He underwent a splenectomy with diagnostic and therapeutic intent. Histopathological evaluation was consistent with a primary splenic CHL. The background spleen showed EMH. His thrombocytopenia improved post-splenectomy.

**Conclusion:**

We report an uncommon example of primary splenic CHL with concurrent ITP and splenic EMH, highlighting the associated diagnostic challenges and reviewing the relevant literature.

Additionally, we hope to emphasize the pivotal role of splenectomy in both diagnosis and management of this patient, as well as explore possible connections between splenic CHL, ITP and EMH.

## Introduction

Classic Hodgkin lymphoma (CHL) is a lymphoma derived from germinal center B-cells and is characterized by neoplastic Reed-Sternberg cells embedded in a reactive microenvironment.^1^ Primary splenic CHL is rare, comprising 0.5% of all splenic lymphomas.^2,3^ Immune thrombocytopenia purpura (ITP) is a well-known complication of B-cell non-Hodgkin lymphoma (HL) but is estimated to affect up to 1% of patients with nodal HL with no prior reports of its affiliation with primary splenic HL.^4,5^ We present a rare example of primary splenic CHL in association with ITP and background splenic extramedullary hematopoiesis. To our knowledge, this is the first report describing this unusual association between these entities, emphasizing the necessity for thorough clinical and pathological evaluation.

## Case Report

**Figure 1.**
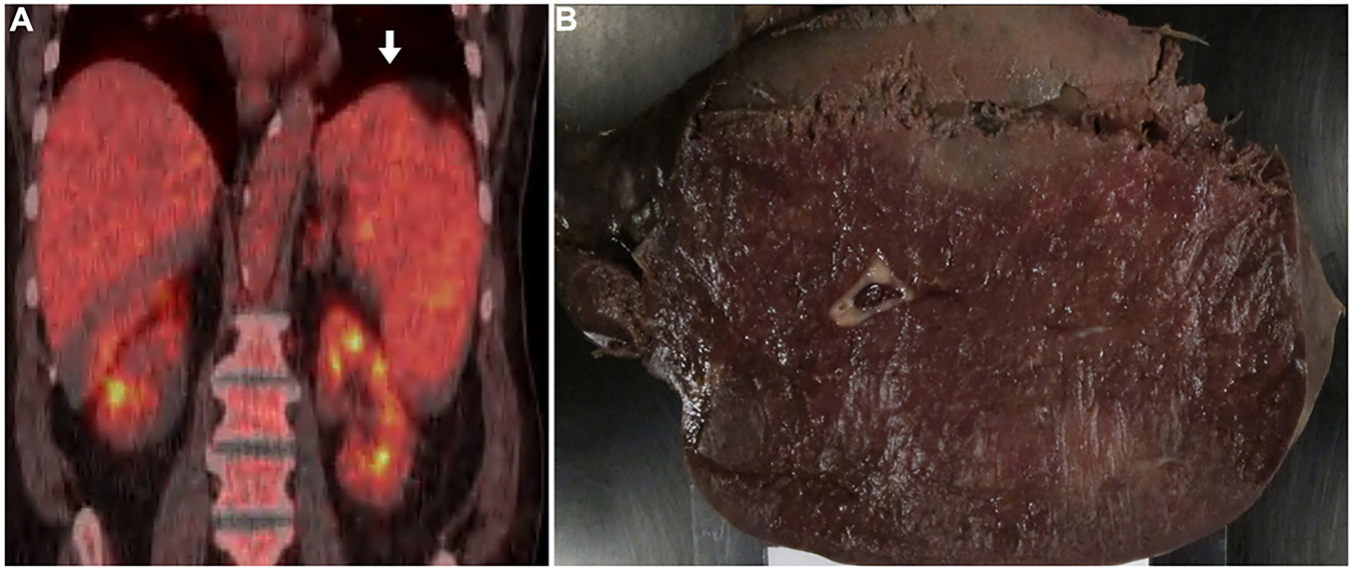
Imaging and gross findings: (A) PET/CT scan showing splenomegaly with diffuse mild homogenous uptake (white arrow). (B) Gross image of the spleen showing splenic congestion with no obvious lesions or masses.

A 70-year-old man with a medical history of atrial fibrillation presented with severe epistaxis and wet purpura in the mouth and platelets of 7 × 10^9^/L. He had noted night sweats and fever for three months prior to presentation and had chronic mild thrombocytopenia (90–110 x10^9^/L). His other complete blood counts at presentation included a hemoglobin of 118 g/L and white blood count of 5.9 × 10^9^/L. Computed tomography (CT) scan showed splenomegaly with scattered hypodensities in the spleen but no lymphadenopathy. A Positron emission tomography CT (PET/CT) scan (Figure 1-A) showed splenomegaly (18 cm) with diffuse homogenous uptake slightly above background liver activity (SUV max 5.6). There were no PET avid lesions or lymph nodes otherwise. The initial clinical suspicion based on imaging results was an indolent lymphoma complicated by ITP. He was treated with IV immunoglobulin, rituximab (one dose), steroid and eltrombopag with no response. He underwent a splenectomy with diagnostic and therapeutic intent. Anticoagulation with rivaroxaban for atrial fibrillation was held prior to splenectomy and not restarted.

**Figure 2.**
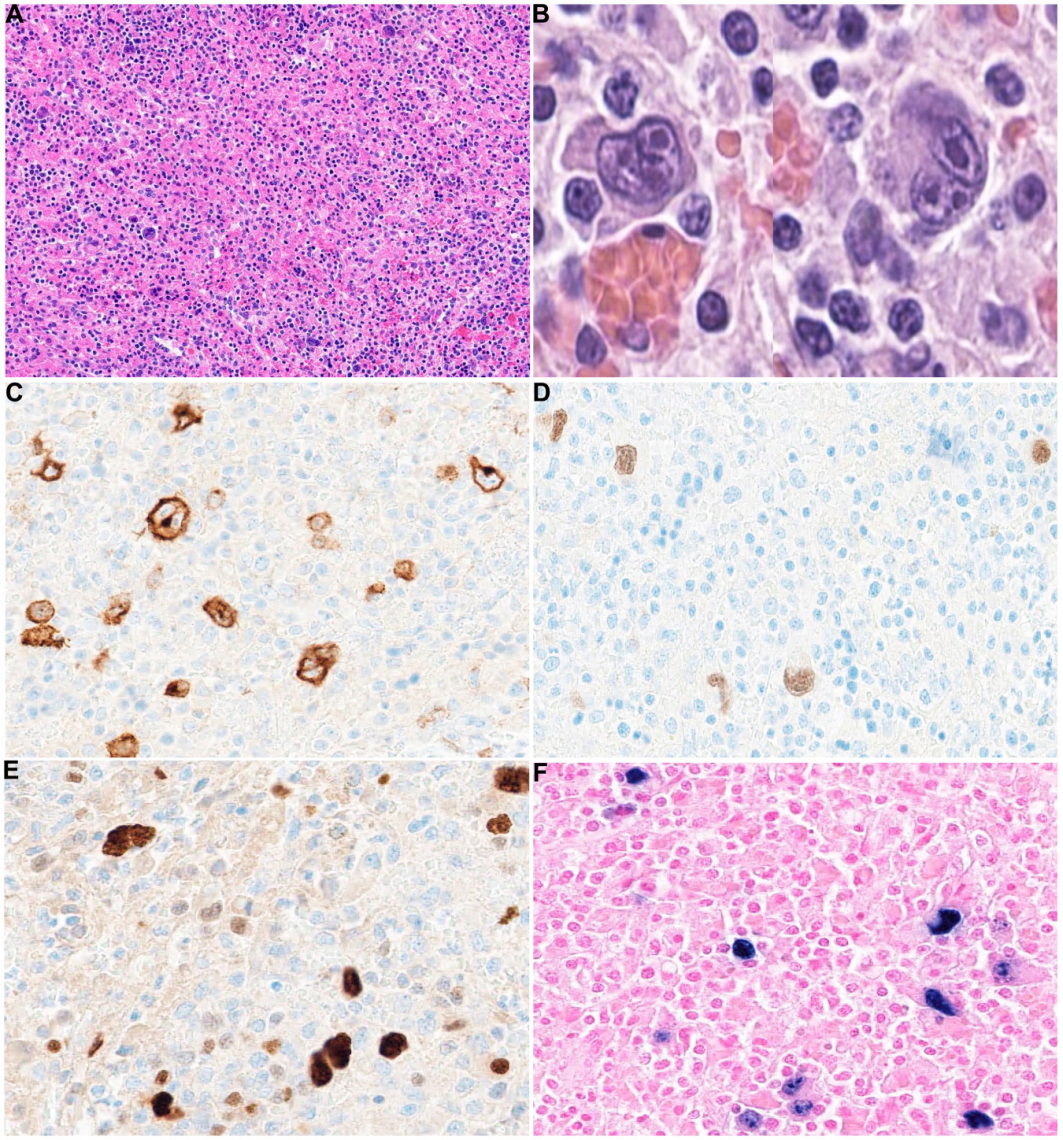
Histopathological and immunohistochemical findings of classic Hodgkin lymphoma in the spleen: (A) low power view of a section of the spleen showing scattered large, atypical cells in a reactive background with mixed inflammatory cells (H&E, 10x). (B) High power view of the Reed-Sternberg (RS) cells with binucleation and prominent macronucleoli (H&E, 40x). The RS cells stained positively for CD30 (C, 20x), weak PAX5 (D, 20x), MUM1 (E, 20x) and EBER (F, 20x).

**Figure 3.**
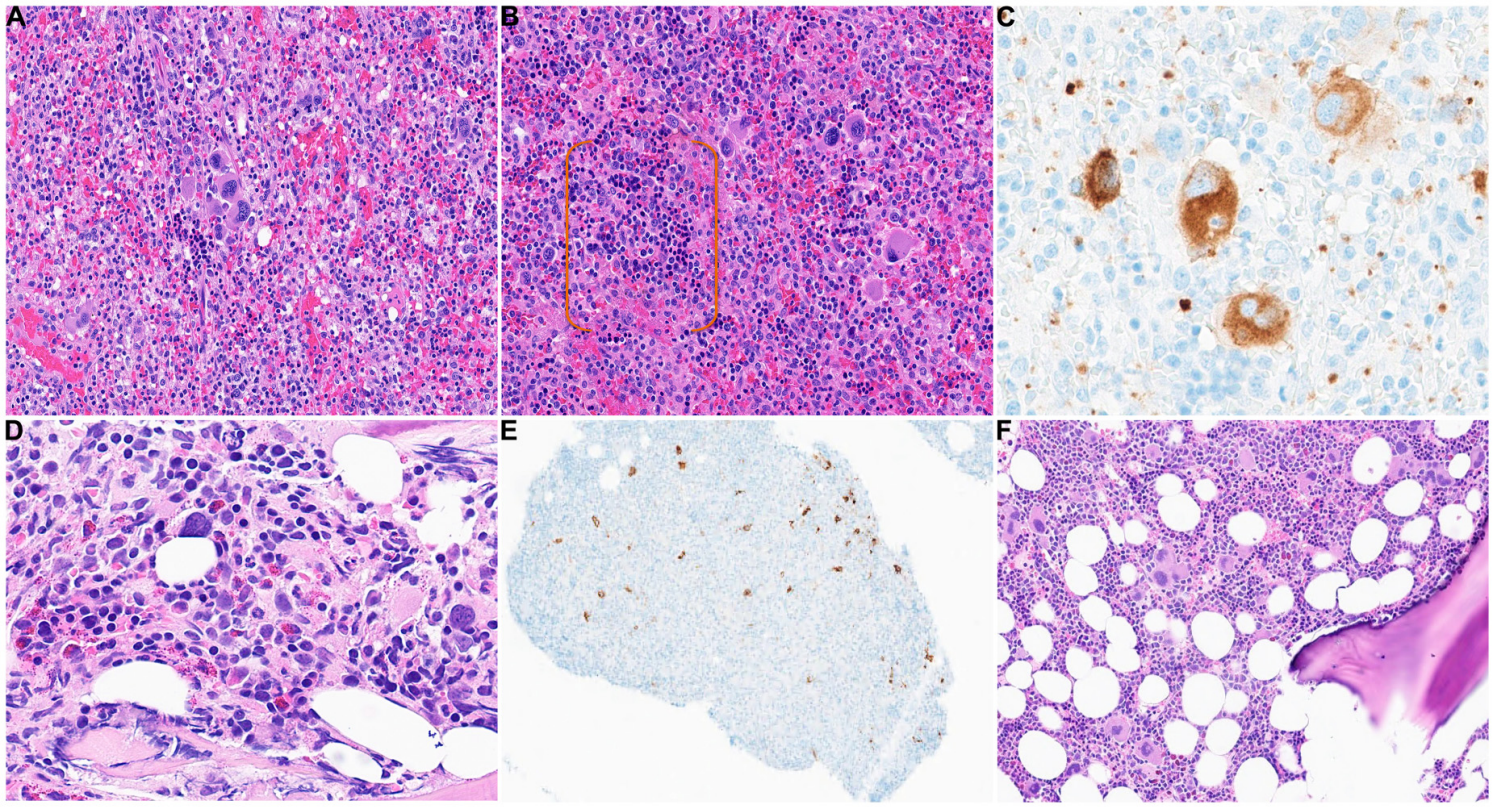
Extramedullary hematopoiesis (EMH) in the spleen and bone marrow findings: (A-B) showing EMH in the spleen with clusters of megakaryocytes and islands of erythroid cells highlighted with the orange brackets in panel (B) (H&E, 20x). (C) Megakaryocytes highlighted by CD61 stain (40x). Panel (D) shows bone marrow involvement by classic Hodgkin lymphoma with scattered large atypical cells (Reed-Sternberg cells) in the background of mixed inflammation with numerous eosinophils (H&E, 40x). (E) The Reed-Sternberg cells in the marrow were positive for CD30 (4x). (F) The background marrow with increased megakaryocytes (H&E, 10x).

Gross examination of the spleen revealed an enlarged spleen (1273.4 g) with no obvious lesions or nodularities (Figure 1-B). Histopathological evaluation revealed patchy areas showing scattered large, atypical cells embedded in a reactive background of lymphocytes, plasma cells and granulocytes (Figure 2-A). These large cells exhibited binucleation and prominent nucleoli consistent with the Reed-Sternberg morphology (Figure 2-B). The atypical cells were positive for CD30 (Figure 2-C), PAX5 (weak) (Figure 2-D), CD15, MUM1 and EBER (Figure 2-E-F), and were negative for CD45, CD79a, CD20 and ALK. The findings were consistent with classic Hodgkin lymphoma. The background spleen showed extramedullary hematopoiesis (EMH) (Figure 3-A-C). The bone marrow was also minimally involved by CHL (Figure 3-D-E). There was no evidence of any other bone marrow pathology to explain the EMH (such as primary myelofibrosis). Additional workup for this patient revealed large platelets in peripheral blood and increased megakaryocytes in the bone marrow (Figure 3-F). These findings have been reported to occur in ITP.^6^ The thrombocytopenia improved post-splenectomy (platelets increased to 91 x10^9^/L and continued to trend upwards). The patient was planned to start brentuximab vedotin and 6 cycles of chemotherapy. Prior to the initiation of treatment, his platelet count rose significantly, and he unfortunately developed a fatal pulmonary embolism. Multiple risk factors may have contributed to the thromboembolic event. These include his post-operative state, prior splenectomy (which likely also contributed to his thrombocytosis), presence of an active malignancy, cessation of anticoagulation therapy and the history of ITP (which could be associated with a paradoxically increased risk of thrombosis).^7,8^

Other investigations performed: flow cytometry was performed on the peripheral blood and bone marrow and showed no evidence of a monoclonal non-Hodgkin B-cell lymphoproliferative disorder. HIV and hepatitis serologies were done and were negative. IgG was performed and was slightly high at 26.6 g/L. Epstein-Barr virus (EBV) Polymerase chain reaction (PCR) was positive at 503 IU/ml. Quantitative PCR for cytomegalovirus was negative. Creatinine, electrolytes, bilirubin, lactate dehydrogenase and liver function tests were all within normal limits. A graphical timeline of the laboratory workup in relation to the patient's treatments are demonstrated in Table (1). Given that therapeutic splenectomy occurred shortly after the patient failed to respond to therapy, antiplatelet antibody testing was not performed; nevertheless, we recognize its utility in ITP, albeit limited in certain contexts.

**Table 1.** Graphical Timelines of Laboratory Workup, Treatments and Outcome.

Laboratory Workup	Treatment		CBC-Post Splenectomy	Staging Marrow	CBC	Outcome
At time of presentation (month 0)	Months 0-2	Month 2	Month 2	Month 2	Month 3	Month 4
**CBC:** – PLT: 7 x10^9^/L – WBC: 5.9 x10^9^/L – Hgb: 118 g/L – MCV: 83.2 fL – ANC: 4.0 x10^9^/L	1. Dexamethasone: 40 mg po x 4 days 2. IVIG: 1 gram/kg x 2 doses 3. Prednisone: 100 mg po daily x 2 weeks 4. Rituximab: 375 mg/m2 weekly x 4 doses 5. Eltrombopag: 75 mg/day x 1 week then 100 mg/day	Splenectomy Rivaroxaban stopped. Mechanical sequential device (SCD) applied for venous thromboembolism prophylaxis	− PLT: 36–94 x10^9^/L – WBC: 9.8–10.7 x10^9^/L – Hgb: 108–113 – MCV: 86.5–88.2 fL – ANC: 7.47 x10^9^/L	Minimal involvement by classic Hodgkin lymphoma	– PLT: 1146–1405 x10^9^/L – WBC: 17.1 x10^9^/L – Hgb: 112 g/L – MCV: 81.3 fL – ANC: 12.1 x10^9^/L	Passed away from pulmonary embolism
**Metabolic workup:** – AST: 20 U/L – ALT: 16 20 U/L – Bilirubin (total): 7.6 unmol/L – LDH: 193 U/L – Creatinine: 63 umol/L – eGFR: >=90						
**Microbiology:** – EBV PCR: 503 IU/ml – EBNA: positive – HIV/Hepatitis serologies: negative						

CBC: complete blood count, PLT: platelets, WBC: white blood count, Hgb: hemoglobin, MCV: mean corpuscular volume, ANC: absolute neutrophil count, AST: aspartate aminotransferase, ALT: alanine aminotransferase, LDH: lactate dehydrogenase, eGFR: estimated glomerular filtration rate, EBV: Epstein-Barr virus, EBNA: Epstein–Barr nuclear antigen, HIV: human immunodeficiency virus.

## Discussion

Our report highlights an unusual presentation of CHL in the spleen with concurrent ITP. While the diagnosis was eventually established based on diagnostic Reed-Sternberg cells and supporting immunohistochemistry, it was a challenging diagnosis primarily due to the rarity of CHL in the spleen, the absence of lymphadenopathy elsewhere, the lack of any gross pathological findings, the patchy distribution of disease within the spleen and the extensive EMH with numerous megakaryocytes in the background.

Primary splenic lymphomas are rare comprising of 1% of all lymphomas, with the vast majority being non-HL.^9^ While nodal HLs can affect the spleen, primary splenic HL exclusively involve the spleen and surrounding hilar lymph nodes without evidence of peripheral lymph node involvement.^9,10^ As noted earlier, primary splenic HL account for a very small proportion of all splenic lymphomas (0.5%).^2^ Both CHL and nodular lymphocyte predominant HL have been described to occur in the spleen.^11^ Several variants of CHL have been reported in the spleen including lymphocyte-depleted CHL and nodular sclerosis CHL.^3,10^ Parekh et al described an example of lymphocyte-depleted CHL in the spleen and its association with an EBV positive status.^3^ It is interesting that our tumor was also EBV positive.

Clinically, splenic HL typically presents with constitutional symptoms and abdominal distention, and complications of the disease can include splenic rupture.^10^ On radiology, several findings can be present including splenomegaly and hypodense splenic lesion/s.^3,10^ Gross examination of the spleen typically shows firm nodules or mass/es which contrasts with what we observed in our tumor.^3,10,12^

Both primary splenic CHL, and CHL in association with ITP are quite rare (<1%) based on previous studies.^5^ The link between CHL and ITP is still unclear and warrants further investigation into the possible mechanisms behind it.^5,13^ HL is recognized as a highly dynamic lymphoproliferative disorder characterized by various pathogenic mechanisms that enable it to modulate the immune system and promote its own proliferation.^13^ Notable mechanisms include its association with Epstein-Barr (EBV) and immunodeficiency states, as well as its capacity to evade the immune system.^13^ It is plausible that the underlying mechanism of ITP in these patients may be linked to the manipulation of the regulatory T-cell activity, which plays a crucial role in autoimmunity and possibly leading to autoimmune destruction of platelets.^13^ The destruction of platelets may be also a consequence of the splenomegaly associated with the lymphoma. The EBV positivity in the neoplastic cells, as observed in our patient and the previously mentioned report, suggests that EBV infection may act as another contributing factor in the development of thrombocytopenia, potentially also via autoimmune pathways.^14^ A previous study identified a higher prevalence of positive EBV infection by immunohistochemistry in splenectomy specimens from ITP patients compared to controls lending support to this hypothesis.^15^

While CHL can occasionally involve the bone marrow in 3%–18% of nodal CHL, bone marrow involvement has not been reported previously in the setting of primary splenic CHL.^16^ Cytopenias occur in about 32% of patients with bone marrow involvement, with anemia and thrombocytopenia being the most common.^16^ Another study by Laurent et al showed cytopenias in the majority (93%) of CHL involving the bone marrow.^17^ This study also showed EBV positivity in 46% of patients.^17^ In the absence of other etiologies to explain the cytopenias in our patient, they are most likely attributable to marrow involvement by CHL.

EMH in this patient can cause some diagnostic challenges as megakaryocytes are large and multinucleated, and can somewhat resemble Reed-Sternberg cells. However, highlighting them with the CD61 stain and their negative CD30 expression was helpful in distinguishing them from Reed-Sternberg cells. The presence of EMH in this patient also posed some questions regarding its etiology. Hematopoiesis typically occurs in the bone marrow in healthy individuals.^18^ The presence of EMH should trigger further investigation into possible underlying causes such as bone marrow pathology (eg, primary myelofibrosis), severe anemia or hematological malignancies.^18^ ITP has been rarely linked with EMH (5.5% of patients).^18^ There is currently limited literature on EMH occurring in the context of CHL but there is a case report that highlighted nodal CHL associated with ITP and EMH.^18^ The patient described in this report also had anemia and the spleen was minimally involved by CHL.^18^ Another study showed EMH in one of the 18 patients with marrow involvement by CHL, but that was observed post-therapy.^19^ Further research is warranted to investigate the potential link between CHL and EMH, if such a relationship exists.

Occasionally, ITP can be therapy induced secondary to treatment of CHL but in general, managing the CHL is a suitable approach to resolve the ITP.^13,20^ Our patient was also interesting since the splenectomy was performed for both therapeutic and diagnostic purposes. The patient's platelets normalized after the splenectomy, indicating the surgery's success in treating the ITP. However, it is also plausible to consider that the surgery may have contributed to the management of the patient's ITP by effectively addressing the underlying lymphoma.

Finally, potential challenges may arise when attempting to surgically manage patients with CHL who present with ITP, particularly when it comes to reaching a platelet count threshold suitable for obtaining specimens to establish the diagnosis, and this may lead to delays in managing the lymphoma.^5^

In summary, we describe an exceptionally rare example of primary splenic CHL arising in association with ITP and background EMH raising awareness for this unusual presentation of CHL.
